# A brief exposure to rightward prismatic adaptation changes resting-state network characteristics of the ventral attentional system

**DOI:** 10.1371/journal.pone.0234382

**Published:** 2020-06-25

**Authors:** Louis Gudmundsson, Jakub Vohryzek, Eleonora Fornari, Stephanie Clarke, Patric Hagmann, Sonia Crottaz-Herbette

**Affiliations:** 1 Department of Radiology, Centre Hospitalier Universitaire Vaudois (CHUV), and University of Lausanne, Lausanne, Switzerland; 2 Neuropsychology and Neurorehabilitation Service, Centre Hospitalier Universitaire Vaudois (CHUV), and University of Lausanne, Lausanne, Switzerland; 3 Department of Psychiatry, Hedonia Research Group, University of Oxford, Oxford, United Kingdom; 4 CIBM (Centre d'Imagerie Biomédicale), Dept. of Radiology, Centre Hospitalier Universitaire Vaudois (CHUV), and University of Lausanne, Lausanne, Switzerland; 5 Signal Processing Lab 5 (LTS5), Ecole Polytechnique Fédérale de Lausanne, Lausanne, Switzerland; Mathematical Institute, HUNGARY

## Abstract

A brief session of rightward prismatic adaptation (R-PA) has been shown to alleviate neglect symptoms in patients with right hemispheric damage, very likely by switching hemispheric dominance of the ventral attentional network (VAN) from the right to the left and by changing task-related activity within the dorsal attentional network (DAN). We have investigated this very rapid change in functional organisation with a network approach by comparing resting-state connectivity before and after a brief exposure i) to R-PA (14 normal subjects; experimental condition) or ii) to plain glasses (12 normal subjects; control condition). A whole brain analysis (comprising 129 regions of interest) highlighted R-PA-induced changes within a bilateral, fronto-temporal network, which consisted of 13 nodes and 11 edges; all edges involved one of 4 frontal nodes, which were part of VAN. The analysis of network characteristics within VAN and DAN revealed a R-PA-induced decrease in connectivity strength between nodes and a decrease in local efficiency within VAN but not within DAN. These results indicate that the resting-state connectivity configuration of VAN is modulated by R-PA, possibly by decreasing its modularity.

## Introduction

Prismatic adaptation consists of a brief exposure to prisms, during which the subject points towards visual targets. Under rightward-deviating prisms (R-PA), the first pointing trials overshoot to the right; after 10–15 pointing this error disappears due to visuomotor recalibration. When the prisms are taken off, the immediately following pointing trials overshoot to the left; this error constitutes the “aftereffect” of PA [1].

R-PA has been shown to alleviate neglect symptoms in patients with right hemispheric lesions [2–19], very likely by modulating the attentional system [20]. As described in a series of seminal studies, the attentional system comprises two partially segregated networks, referred to as the ventral and dorsal attentional networks (VAN, DAN), which support exogenous and endogenous orienting of attention, respectively [21–23]. DAN comprises the intraparietal sulcus, the superior parietal lobule and the frontal eye field; within this network voluntary shifts of spatial attention tend to yield greater preparatory activity than stimulus-driven shifts [24]. DAN is symmetrical and carries, in either hemisphere, the representation of the contralateral space [25]. VAN is lateralized, the core region being the right temporo-parietal junction [26]. The connectivity of DAN and VAN has been investigated by dynamic causal modeling of effective connectivity during lateralized orienting and reorienting of attention, based on the Posner paradigm [27,28]. Strong connections were demonstrated within and between the left and right parts of DAN and from the right temporo-parietal junction to VAN. Taken together, current evidence concurs that VAN and DAN are anatomically distinct, albeit interconnected, and that they have distinct functions. Whereas VAN is mainly involved in attention to unattended stimuli, DAN is activated by voluntary top-down attention processing [23].

Pointing under R-PA modulates the activation in the anterior cingulate cortex, the anterior intraparietal sulcus and the cerebellum, yielding greater activity during the early as compared to the late stages of the adaptation [29]. Comparing the activation patterns before and after a brief exposure to R-PA revealed two types of changes. First, the representation of the left, central and right space, in visual and auditory modalities, increased within the left VAN, whereas the representation of the right space decreased in the right VAN, switching thus the hemispheric dominance from the right to the left hemisphere [30,31]. A similar change in VAN lateralization was shown to occur in neglect patients after right hemispheric lesions [30]. Second, tasks known to rely on DAN, such as line bisection or visual search, yielded greater activation within DAN after R-PA, as shown in patients with neglect [32]. Both types of changes in the organization of the attentional system occur very rapidly, over a few minutes, and reflect thus functional reorganization rather than structural changes [20].

The impact of R-PA on resting state connectivity was investigated in normal subject. Using 1.5T fMRI data, Tsujimoto and colleagues performed a seed-based correlation analysis within the attention network and found a significant decrease of connectivity between the right intraparietal sulcus and the right frontal eye field and a significant increase connectivity between the right frontal eye field as well as the right anterior cingulate cortex; this was interpreted as a modulation of DAN within the right hemisphere [33]. In a parallel study, Tsujimoto and colleagues reported a change in resting-state functional connectivity between the primary motor cortex and the cerebellum, which correlated with the amplitude of R-PA aftereffect [34]. Global connectivity was analyzed in a recent 3T fMRI study; R-PA induced a significant decrease in two nodes of the default mode network (DMN) and the left anterior insula. Seed-based post-hoc analysis from these regions showed decrease in connections to parts of the attentional system (the inferior frontal gyrus, the anterior insula and the right superior temporal sulcus) as well as within DMN [35].

The above evidence indicates that R-PA leads to a reorganisation of the attentional system and modulates resting-state connectivity, possibly affecting network configurations. Network science represents brain organization by graphs, which consist of nodes and edges. Nodes are regions or areas, depending on the spatial resolution which is used, edges are connections between nodes [36]. The efficiency of a network describes relationships between nodes and eventually the balance between functional segregation (or local efficiency) and global integration (or global efficiency) [37]. Functional segregation relies on clusters, or subunits, each of which has dense internal connections; they are presumed to share specialized information and be involved in the same task. Functional integration refers to the ease in accessing each point of the graph from any other point, including across subunits. A network with a high local efficiency, i. e., strongly segregated, is believed to rely on small subunits to execute specific tasks. Conversely, a network with high global efficiency, i. e., strongly integrated, tends to work as a whole.

Network characteristics of the human brain, as defined on the basis of structural and functional connectivity, combine high local and high global efficiency and involve network communities, often referred to as modules, and connecting hubs [38,39]. In network science a module is defined as a subnetwork that is composed of densely interconnected nodes and is only relatively sparse connected to the other parts of the networks [40]. Dense local connections, believed to be characteristic of modules, have been demonstrated in human post-mortem tracing studies in primary visual [41] and auditory cortices [42] as well as in higher-order visual areas [43], temporal cortex [44] and Broca’s area [45]. Modularity, as revealed in vivo by functional connectivity, is malleable. In a recent study, attentional networks were shown to increase their inter-module connectivity when confronted with a challenging cognitive task. In terms of efficiency this resulted in a lesser segregation and thus a putative loss of modularity [46,47].

We made use of network science [48] to analyze the effects of prismatic adaptation on the configuration of the attentional system. Our working hypothesis was based on the observation that R-PA induces a rapid reorganization of the attentional system, including the emergence of ipsilateral spatial representation within the left VAN [30,49,50] and changes in responsiveness within DAN bilaterally [32]. Both phenomena are likely to rely on changes in functional connectivity, which appears to be modulated within DAN [33] as well as within VAN and DMN [35].

We hypothetized that a brief exposure to R-PA leads to a decrease in local efficiency and thereby a loss of modularity, in analogy to that of challenging cognitive tasks [46,47]. Because of the major functional re-organization, which R-PA induces within VAN [49,30,50,20], we expected R-PA related changes in modularity within VAN.

To test this hypothesis we compared resting-state connectivity before and after a brief exposure to R-PA with i) a whole brain approach and ii) focused approach on the attentional networks VAN and DAN. The latter part involved three measures of network efficiency [37,51], namely i) *connectivity strength within a network* (calculated as the average correlation between the nodes of the network); ii) *global efficiency*, reflecting the level of functional integration and inversely related to the average shortest path between each pair of nodes; and iii) *local efficiency*, reflecting functional segregation and inversely related to the average path length between the neighbors of a node.

## Materials and methods

### Subjects

Twenty-six subjects participated to this study, 14 participants in the R-PA group (7 men, mean age = 26.0 years, SD = 5.0 years) and 12 in the control group (6 men, mean age 25.8 years SD = 4.8 years). All subjects were right handed and all had a normal or corrected-to-normal vision. None reported history of psychiatric or neurological disease. The experimental procedures were approved by the Ethics Committee of the Canton de Vaud; all subjects gave written, informed consent. Subjects in both groups were scanned two times, before (timepoint 1) and after (timepoint 2) a brief pointing task while wearing prisms (R-PA) or plain glasses (control).

### Visuomotor adaptation task

Participants in the R-PA group wore glasses mounted with prism lenses with a 10° rightward deviation, while the control group wore plain glasses. Both groups were instructed to point with their right index finger at two targets located in the right or left side of the participants (14° on the right or on the left of the participants’ mid-line) during three minutes corresponding to approximately 150 pointing [31,49]. Subjects did not see the first half of the pointing movements. Experimenter indicated verbally to the subjects which target they had to point. To maintain subjects attentive to the pointing movements, the experimenter varied the pace of his verbal indications and encourage the subjects to do quick movements. After the removal of the prisms, we measured the visuomotor deviation by instructing the subjects to close their eyes and point to the left or right targets. Participants in the R-PA group showed errors in the opposite direction of the prism deviation when pointing with their eyes closed; this is the expected “after-effect” of the R-PA. We measured this after-effect by the average of pointing distance from the targets in mm (positive values for right, negative values for left). The number of measures were limited to avoid de-adaptation. The magnitude of the behavioral aftereffect yielded by R-PA was analyzed by two-way mixed model ANOVA with the within-subject factor Side of target (Left, Right) and the between-subject factor Group (R-PA, control), using SPSS (version 23).

### Data acquisition

Data were acquired on a 3T Siemens Magnetom Trio scanner with a 32-channels head-coil at the Lemanic Biomedical Imaging Center (CIBM) in the CHUV, Lausanne. Each participant underwent three MRI acquisitions: 1) T1-weighted 3D gradient-echo sequence (160 slices, voxel size = 1 x 1 x 1 mm), 2) diffusion spectrum imaging sequences (DSI) (257 diffusion-weighted volumes, 1 b0 volume, maximum b-value 8000 s/mm^2^, resolution of 2.21 × 2.21 × 3 mm) and 3) a resting-state functional MRI (single-shot echo planar imaging gradient echo sequence) of 10 minutes (voxel size = 3 x 3 x 3 mm, repetition time = 2 s; echo time = 30 ms; flip angle = 90°; number of slices = 32; 10% gap). In the fMRI sequence, the 32 slices were acquired in a sequential ascending order, and covered the whole head volume in the AC-PC plane. We limited head movements with head’s paddings. Furthermore, subjects were scanned twice, before (timepoint 1, tp1) and after (timepoint 2, tp2) the visuomotor adaptation and were instructed to relax without thinking about anything in particular and to keep their eyes open. Several task-related functional acquisitions were done but not analyzed here [49].

### Computation of the connectivity matrices

Using the connectome mapper software (an Open-Source Processing Pipeline to Map Connectomes with MRI) and Matlab, we obtained the structural and functional connectivity matrix for each subject and timepoint based respectively on DSI tractography and BOLD fMRI data.

### Computation of structural connectivity matrices

**Fig 1:**
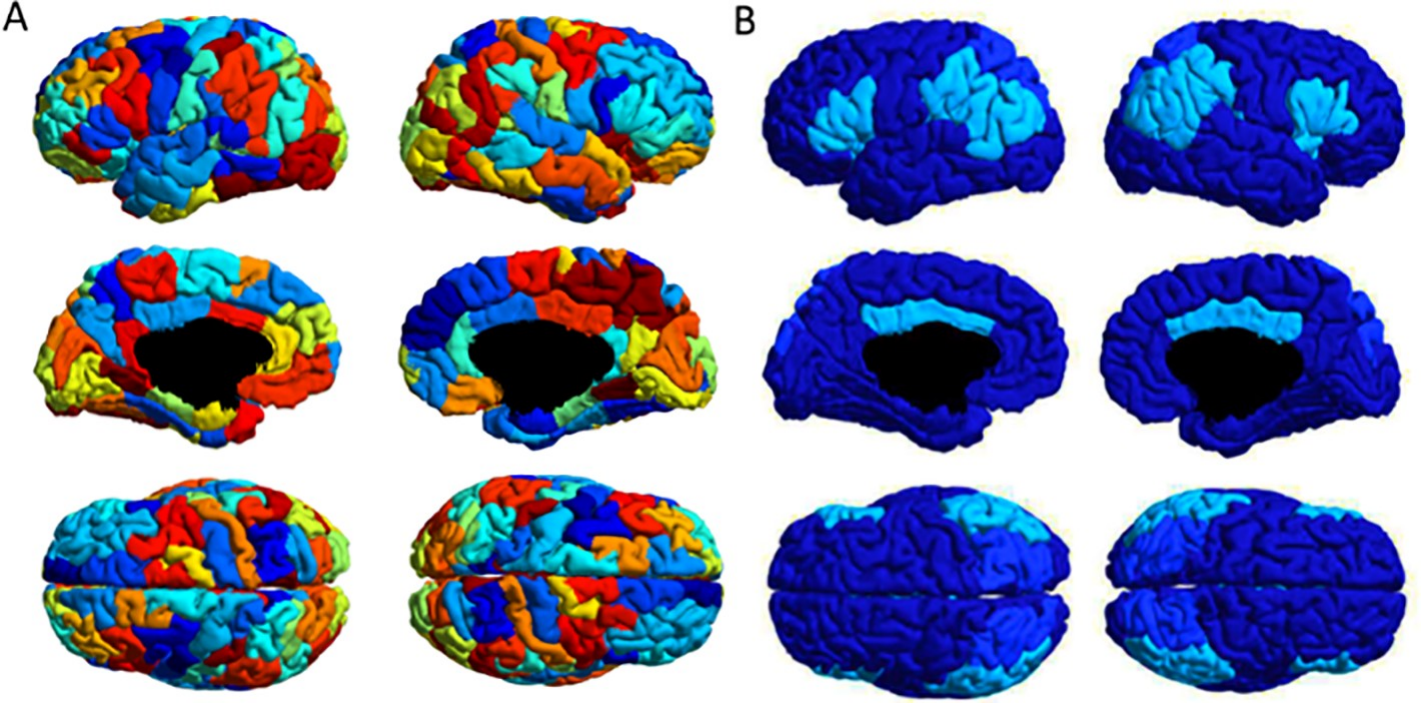
A. Cortical parcellation into 129 ROIs [53]. B. VAN and DAN as defined by Yeo et al. [60]. The regions are from lighter to darker, cyan blue: ROIs included in the VAN, azure blue: ROIs included in the DAN, marine blue: rest of the brain.

For each subject and timepoint the T1-weighted image was used to segment the grey and white matter in Freesurfer [52], implemented through the connectome mapper pipeline. We then parcellated the cortex into 129 regions of interest (Fig 1) with the “Lausanne 2008” parcellation [53] implemented in Freesurfer. The average cortical surface area of cortical ROIs was 18 cm^2^ (SD 7.5 cm^2^). Furthermore, we registered diffusion volumes to T1 data, realigned the volumes and corrected head motion with the FMRIB's Linear Image Registration Tool (FLIRT) [54,55].

For diffusion spectrum imaging (DSI) tractography, we used a streamline deterministic fiber tracking algorithm with 32 seeds per voxel of white matter, the tracks below 8 or above 500 fibers were considered as noise, the stopping criteria was an angle above 60°. We then generated the individual structural connectivity matrices with the function integrated in the connectome mapper [56].

### Computation of functional connectivity matrices

The resting state connectivity matrix of each subject and timepoint was computed with the connectome mapper and Matlab as follows. T1-weighted segmentation and parcellation used the same settings as for DSI data. We registered resting state fMRI volumes to T1 data and corrected head motions with FLIRT [54,55]. Then we conducted a slice timing correction to temporally realign the slices of each resting state fMRI volume. A nuisance regression was computed using linear regression with the average signal of white matter and cerebrospinal fluid. To address thermal effects, we conducted a polynomial detrending. For each resting state fMRI scan, the first five of 242 acquisitions were discarded to use only the series obtained after stabilization of the MRI scanner. We continued the preprocessing with Matlab toolboxes and homemade Matlab scripts for low and high pass frequency filtering (0.01 and 0.1 Hz). We then applied a Fischer Z transform on the Pearson correlation matrices to allow group comparisons with the assumption of normally distributed data.

### Computation of mixed structural and functional connectivity matrices

With the data of the individual structural connectomes of both groups we computed a binary structural connectome, referred to as group representative structural connectivity matrix, where each intersection was assigned a 1 if a connection was present in at least 50% of the 26 subjects and 0 otherwise. This approach was used to minimize false positive and false negative white matter connections, as described in previous publications by others [57]. Including both groups together to compute the group representative connectivity matrix allowed to create a unique template of white matter connections that was used in both groups. Thus, putative differences in structural connectivity between the two groups could not induce structural biases to the functional connectivity analyses. The representative structural connectivity matrix was used to filter the individual functional connectivity matrices to obtain a mixed structural and functional connectivity matrix for each subject and timepoint [57]. In the mixed matrix of each subject and timepoint, each cell was equal to the value of the corresponding cell in the individual functional connectivity matrix when a structural connection existed in the representative structural connectivity matrix. If there was no structural connection in the representative structural connectivity matrix, the value in the individual mixed matrix was equal to zero. Therefore, these mixed matrices were specific for each subject and each timepoint.

### Comparison of whole brain functional connectivity

We conducted a mixed two-way ANOVAs with the within subject factor Timepoint (tp1, tp2) and the between subject factor Group (R-PA, control) on the functional connectivity matrices. To correct for multiple comparisons, the ANOVA was computed with the false discovery rate method [58] by using the Network Based Statistics toolbox (NBS) [59]—a statistical toolbox which identifies differences in brain networks.

### Comparison of the connectivity of functional networks

In another analysis, we focused on two resting state networks, VAN and DAN, as defined by Yeo and colleagues [60]. After parcellating the cortex into 129 regions of interest (whose mean surface area was 18 cm^2^, SD 7.5cm^2^) [53], we have identified regions of interest belonging to the attentional networks [60]. VAN consisted of 19 ROIs, 10 in the right and 9 in the left hemisphere, of which three were in the right and two in the left inferior parietal lobule respectively; the average surface area was 18.5 cm^2^ (SD 6.5cm^2^). DAN consisted of 6 ROIs, three in each hemisphere, with an average surface area of 18.5 cm^2^ (SD 3.7cm^2^).

Network science approach was applied to the connectivity analysis of VAN and DAN. For each network, subject and timepoint separately, we determined measures of functional connectivity matrices as defined in the brain connectivity toolbox of Rubinov and Sporns [51]. We applied two main types of measures, the first type was based solely on the functional connectivity matrices, the second type, computed on the mixed connectivity matrices, integrated the structural connectivity information obtained from fiber tractography. First, the average correlations between the nodes inside the functional system submatrix, without taking connections to the outside nodes into account, were calculated. We refer to this first measure as *connectivity strength within network*. Second, to determine if the changes in our connectivity measures were more influenced by changes in functional integration or functional segregation of our networks we calculated the weighted *global efficiency* and weighted *local efficiency* that respectively reflect integration and segregation [51]. Local efficiency and global efficiency measures are based on path lengths and are therefore computed on the basis of a mixed structural-functional connectivity matrices (functional connectivity matrices alone do not represent information flow but statistical correlations [51]).

Connectivity strength, global efficiency and local efficiency were analyzed within VAN and within DAN, each of them analyzed with a mixed 2-way ANOVA with the factors Timepoint (tp1, tp2) and Group (R-PA, control) using SPSS.

## Results

### Behavioral aftereffect of a brief exposure to R-PA

The aftereffect, i. e., the pointing error that occurred during the first trial after the prisms (or plain glasses in the control group) were removed, was -66± 16 mm (mean ± SD) for left and -56 ± 19 for right targets in the R-PA group and +5 ± 10 mm for left targets and +6 ± 8 for right targets in the control group. Two-way mixed model ANOVA with the within-subject factor Side of target (Left, Right) and the between-subject factor Group (R-PA, control) yielded a significant main effect of group (F(1,24) = 141.38; p<0.001), driven by larger aftereffect in the R-PA than the control group.

### R-PA-induced changes in whole brain functional connectivity

**Fig 2:**
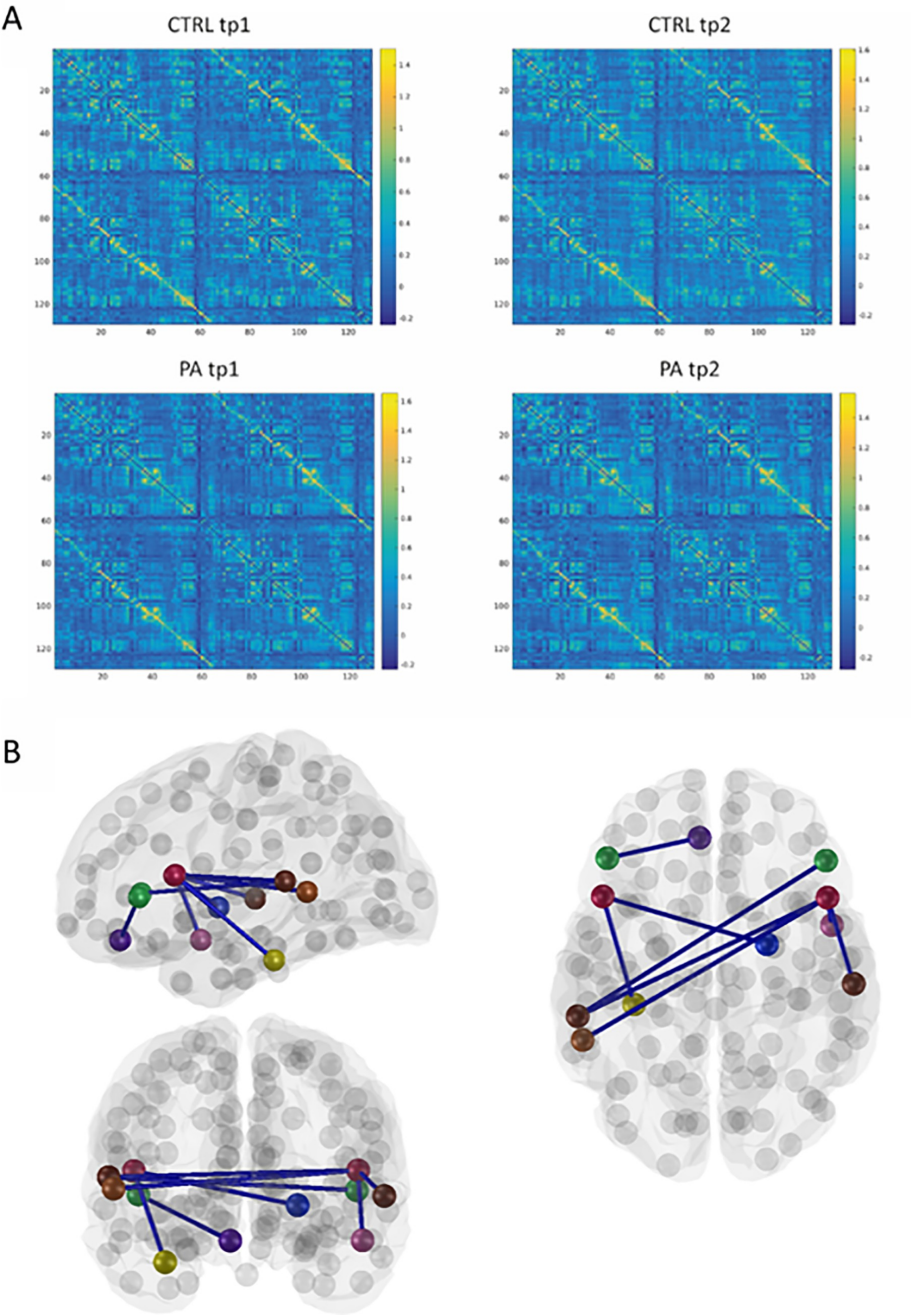
A. Resting state connectivity matrices before (left; tp1) and after (right; tp2) visuo-motor adaptation for the R-PA group (PA) and for the control group (CTRL). B. Brain figures highlighting edges that yielded a significant R-PA-related decrease in connectivity (as demonstrated by a significant interaction Group x Timepoint and a significant post-hoc analysis (Table 1). Color code designates nodes: red: IFG pars opercularis; green: IFG pars triangularis; brown: STG posterior part; pink: STG anterior part; light brown: STS posterior part; yellow: FG anterior part; purple: OFG medial part. Same abbreviations as in Table 1.

A resting state connectivity matrix was calculated for each subject and timepoint (Fig 2A). Each of the individual cells of the matrices were compared with a mixed 2-way ANOVA with between subject factor Group (R-PA, control) and within subject factor Timepoint (tp1, tp2). Significant interaction (F(1,24) = 6.9; p<0.05) were found in 11 cells, corresponding to a bilateral, fronto-temporal network, which consisted of 13 nodes and 11 edges. Apart from this fronto-parietal network, no other cells yielded significant interaction Group x Timepoint.

Post-hoc t-tests were carried out on the connections that showed significant interaction Group x Timepoint. Out of the 11 edges, which yielded a significant interaction, eight had a significantly lower functional connectivity at timepoint 2 than at timepoint 1 in the R-PA group (paired t-test t(13) = 2.34; p<0.05) but not in the control group (Table 1). Three edges were within the right hemisphere and linked nodes within the inferior frontal gyrus with nodes in the superior temporal gyrus (Fig 2B). Two edges were within the left hemisphere and linked the inferior frontal gyrus with either the orbitofrontal gyrus or the fusiform gyrus. Three edges were interhemispheric and linked nodes within the right inferior frontal gyrus with nodes on the left superior temporal gyrus or sulcus. An additional interhemispheric edge linked the right pallidum with a node in the left inferior frontal gyrus.

**Table 1:** Edges, i. e. connections between two nodes, that displayed a significant interaction Group x Timepoint (FDR corrected; left and middle columns). Post-hoc analysis (paired t-test, p < 0.05) yielded in the R-PA group significant difference for eight of them, driven by lower connectivity at timepoint 2 than at timepoint 1 (right column). No significant differences were found in the CTRL group. IFG: inferior frontal gyrus; FG: fusiform gyrus; OFG: orbitofrontal gyrus; STG: superior temporal gyrus; STS: superior temporal sulcus; TG: transverse gyrus; R: right; L: left.

Node 1	Node 2	Post-hoc comparison tp2 vs. tp1, R-PA group
R IFG pars opercularis	R STG posterior part	p = 0.040
R IFG pars triangularis	R STG posterior part	not significant
R IFG pars opercularis	R STG anterior part	p = 0.049
R IFG pars opercularis	R TG lateral part	not significant
L OFG medial part	L IFG pars triangularis	p = 0.006
R Pallidum	L IFG pars opercularis	p = 0.015
L IFG pars opercularis	L FG anterior part	p = 0.009
R IFG pars opercularis	L STS posterior part	p = 0.016
R IFG pars triangularis	L STG posterior part	p = 0.045
R IFG pars opercularis	L STG posterior part	p = 0.038
R IFG pars opercularis	L TG lateral part	not significant

All edges, which displayed a significant interaction Group x Timepoint, involved one of four nodes within the inferior frontal gyrus, two in the right and two in the left hemisphere. The inferior frontal region is generally considered as part of VAN [60]. Thus, the whole-brain analysis indicates that VAN and networks related to it are modulated by R-PA.

### R-PA-induced modulation of the attentional networks

Whole brain analysis highlighted R-PA-induced modulation of the connectivity of VAN, leading possibly to changes in network efficiency. We have analyzed this latter hypothesis by means of three measures of network efficiency: connectivity strength; global efficiency; and local efficiency. The same analysis of network efficiency was carried out separately for DAN.

#### Connectivity strength within networks

**Fig 3:**
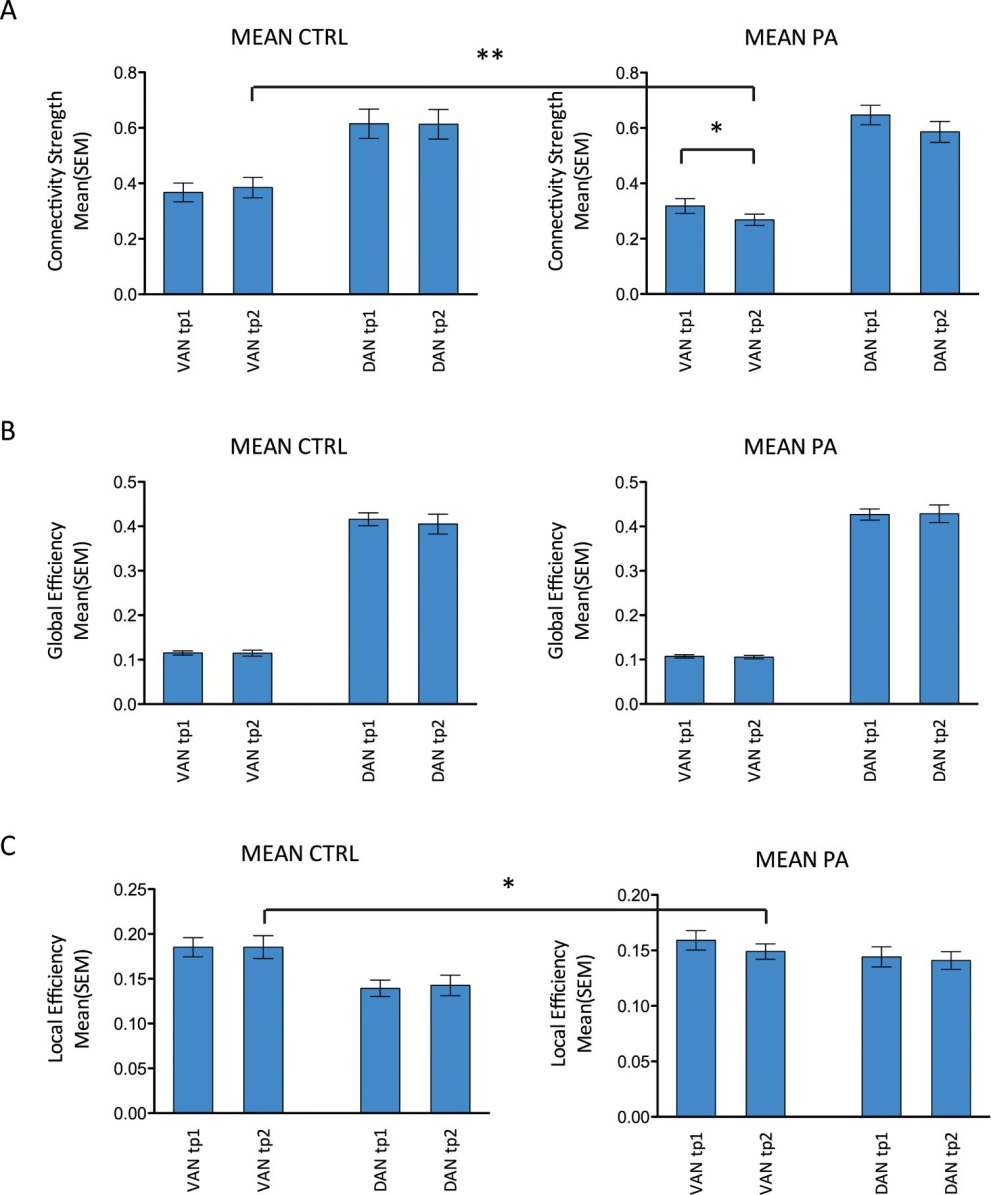
Comparison of connectivity characteristics before and after visuo-motor adaptation for the R-PA group (PA) and for the control group (CTRL) within VAN and DAN. Mean (± SEM) of Connectivity strength (A), Global efficiency (B) and Local efficiency (C). Unpaired t-test between R-PA and CTRL groups, paired t-test between tp1 and tp2 for each group separately (*p ≤ 0.05, **p ≤ 0.01).

Connectivity strength is an index of connections between the nodes of a given network and is expressed as the average correlation between the nodes. Within VAN the 2-way ANOVA with the factors Timepoint (tp1, tp2) and Group (R-PA, control) yielded a significant main effect of Group (F(1,24) = 5.93: p = 0.023), but not a significant interaction or main effect of Timepoint. Post-hoc t-tests carried out for VAN yielded significantly lower connectivity strength at timepoint 2 than at timepoint 1 (paired t-test t(13) = 2.34; p = 0.036) in the R-PA group, whereas no significant difference was present in the control group (Fig 3A); at timepoint 2 connectivity strength was significantly lower in the R-PA than in the control group (unpaired t-test t(24) = 2.91; p = 0.008). No significant main effect nor interaction were present in DAN.

These results indicate that R-PA tended to decrease connectivity strength between the nodes within VAN.

#### Global efficiency of mixed structural-functional connectivity matrices

Global efficiency is a measure of overall exchange of information across a network and reflects functional integration. It corresponds to the average shortest pathway between each pair of nodes of the network. High global efficiency corresponds to efficient connectivity, i.e., short pathways between nodes. The 2-way ANOVA with factors Group and Timepoint was carried out for VAN and DAN separately; it did not yield any significant main effect or interaction in either network.

#### Local efficiency of mixed structural-functional connectivity matrices

Local efficiency is a measure of information exchange at local level, i. e. between direct neighbors, and reflects functional segregation. It is based on the average path length between nodes in the neighborhood of a node. High local efficiency corresponds to low path length. Within VAN the 2-way ANOVA with factors Timepoint (tp1, tp2) and Group (PA, control) yielded a significant main effect of Group (F(1,24) = 7.99; p = 0.009), but not a significant interaction or main effect of Timepoint. Post-hoc t-tests for VAN yielded at timepoint 2 a significantly lower local efficiency in the R-PA than in the control group (unpaired t-test (t(24) = 2.61; p = 0.016). No significant main effect nor interaction was present in DAN.

These results indicate that R-PA tended to decrease local efficiency of information exchange within VAN but did not affect DAN.

## Discussion

Our results indicate that a brief exposure to R-PA induces changes in resting-state connectivity that is primarily present within VAN and that is compatible with a decrease in modularity.

### R-PA-induced reorganization of the attentional system

A series of influential studies have documented the effect of R-PA on visuo-motor plasticity and the ensuing modulation of the attentional system [61–63]. Changes in resting state connectivity, as induced by a brief exposure to R-PA, have been demonstrated in normal subjects with different methodological approaches. Seed-based connectivity analysis demonstrated a decrease of connectivity between the posterior and anterior parts of DAN in the right hemisphere [33]. Comparison of global connectivity of individual nodes highlighted a decrease of connectivity of parts of the default mode network [35]. Network science analysis of connectivity matrices revealed here a decrease of connectivity strength and of local efficacy within VAN and in particular in its anterior part, indicative of a decrease of modularity. The decrease in resting state connectivity coincides with the emergence of ipsilateral visual and auditory spatial representation within the left inferior parietal lobule [49,50]. The decrease in local efficiency within VAN, which we describe here, is indicative of a loss of modularity within VAN and may be instrumental in bringing up the new spatial representation.

A decrease in modularity within VAN and the shift in hemispheric dominance may contribute to the R-PA effect in neglect. As shown in a series of seminal studies R-PA tends to alleviate neglect symptoms [2–19,64], predominantly in patients with anterior lesions in whom the posterior part of the corpus callosum and the right DAN are spared [50,65,66]. Activation studies have demonstrated that R-PA induces in neglect patients a shift of dominance by establishing ipsilateral spatial representation within VAN on the left side [30] and that it increases responsiveness of DAN [32]. Thus, there are similarities in R-PA induced effects between normal subjects and patients with neglect, but several aspects have not yet been investigated. It is currently unknown whether R-PA has the same effect on resting state connectivity in neglect as it has in normal subjects. There may be differences, since focal brain damage is generally associated with wide-spread changes within the ipsi- and contralesional hemisphere, including transmitter receptors [67,68]. These changes are accompanied with changes in the configuration of specialized networks, as demonstrated e. g. by the loss of parallel processing in the contralesional hemisphere [69].

### Rapidity of the R-PA induced modulation

The change in resting state connectivity occurred rapidly, within minutes of the exposure, about in parallel to the effect on visual spatial representation within VAN [20,49]. The rapidity of the effect suggests that it consists of uncovering pre-existing spatial representations within left VAN. The left hemisphere is known to support two whole space representations, which are likely candidates for the effect of R-PA. One bears on visual space and has been demonstrated in a study comparing uncued vs. cued targets in a multi-target environment [70]. The other concerns spatial attention to motricity and has been revealed in relation to the preparation and the redirection of movements and movement intentions [71,72]. Regions of interest as used in our study, i.e., with an average surface area of 18 cm^2^, are within the range of magnitude of clusters revealed in paradigms of multi-target environment [70], motor attention [71,72] or R-PA-induced representation of the ipsilateral space [49,50].

The connectivity changes, which are highlighted by network analysis, concerned connectivity strength and local efficiency in VAN and, as revealed by whole brain analysis, they implicated in particular the frontal part of VAN. The actual axonal connections that are likely to be involved remain to be determined, but there are several candidates. Anatomical studies in human post-mortem material revealed monosynaptic connections from the visual areas in the right inferior temporal cortex to the contralateral, left inferior parietal lobule, superior temporal gyrus, supratemporal plane and the inferior frontal gyrus [73]. It is interesting to note that the inferotemporal region, where this projection originated, is known to carry the representation of the left visual field [74]. These long-distance monosynaptic connections cannot be changed as rapidly as the demonstrated effect of R-PA. As demonstrated in non-human primates long cortico-cortical connections have wide-spread terminal arborizations, which contact a variety of cortical neurons and modulate thus excitatory and inhibitory intracortical circuits [75]. As investigated with anterograde and retrograde tracers in post-mortem tissue, the intrinsic connectivity is particularly spread in the human postero-inferior frontal cortex [45] and at the parieto-temporal junction [44], more so than in early-stage visual or auditory areas [41,42]. Thus, it is likely that the neural basis of the R-PA effect involves an interplay between long cortico-cortical and local intracortical connections. It is interesting to note that in normal subjects, without an exposure to R-PA, the crossed visual occipital to temporo-parietal connections are functionally asymmetrical; shifts of exogenous visual attention were shown to lead to increased effective connectivity between the right temporo-parietal junction and ventral occipital areas on either side, whereas it strengthened only the ipsilateral connection between the left temporo-parietal junction and ventral occipital areas [76].

The rapidity of the R-PA induced change is in keeping with other known changes in cortical sensory representations. A striking example are the receptive fields in monkey somatosensory area 3b; a brief anesthesia of the collateral nerves of the index leads within minutes to the expansion of the receptive fields of the corresponding cortical neurons, which started to respond to large parts of other hand parts [77].

### Plasticity of resting-state connectivity

A brief exposure to R-PA modulates resting-state connectivity [33–35] and was shown here to predominantly decrease the modularity within VAN. The brevity of the R-PA intervention, contrasts with studies of longer exposure to other therapeutic interventions.

Changes in resting state connectivity were reported following other therapeutic interventions, which were administered over several weeks. A first study investigated the impact of a one month training of Attentional Bias Modification, known to alleviate anxiety by moderating attentional processing of threat stimuli. In normal subjects this training, but not an equivalent control condition, enhanced functional connectivity between the pulvinar and the temporo-parietal junction and decreased that between the posterior cingulate gyrus and the anterior insula plus ventrolateral prefrontal cortex [78]. A second study investigated the impact of an intensive 10 weeks reading intervention training program on the reading network. Children with autism spectrum disorders, who underwent the training, improved significantly reading comprehension, in parallel to an increase in local brain connectivity within the reading network [79].

The decrease in modularity within VAN, which we observed here after a brief exposure to R-PA, may be linked to the previously described emergence of the ipsilateral spatial representation in the left VAN [49]. The putative relationship between the decrease of modularity and the shift of hemispheric dominance within VAN is intriguing and may represent a new concept in plasticity. Previous studies have shown a reverse tendency, greater modularity being associated with better responses to cognitive training, both in normal subjects [80,81] and in brain-damaged patients [82]. Furthermore, the re-emergence of a modular organization appears to correlate with recovery after stroke [83].

## Conclusions

A brief exposure to a single session of R-PA led to changes in resting state connectivity. Graph theoretical analysis highlighted significant decrease in connectivity strength and in local efficiency within VAN, which is indicative of a loss of modularity. Thus, in normal subjects these changes are likely to favor the previously described emergence of ipsilateral visual representation within the left inferior parietal lobule and the ensuing shift in hemispheric dominance within VAN [49,50]. Further studies are needed in neglect patients to establish whether in this population changes in resting state connectivity accompany R-PA induced shift in hemispheric dominance [30].

## Abbreviations

DAN: dorsal attentional network
PA: prismatic adaptation
R-PA: rightward prismatic adaptation
tp1: timepoint 1 = before R-PA
tp2: timepoint 2 = after R-PA
VAN: ventral attentional network
